# The shift between the Red Queen and the Red King effects in mutualisms

**DOI:** 10.1038/srep08237

**Published:** 2015-02-04

**Authors:** Lei Gao, Yao-Tang Li, Rui-Wu Wang

**Affiliations:** 1 School of Mathematics and Statistics, Yunnan University, Kunming, Yunnan. 650091, P.R. China; 2 State Key Laboratory of Genetic Resources and Evolution, Kunming Institute of Zoology, Chinese Academy of Science, Kunming, Yunnan. 650223, P.R. China

## Abstract

Interspecific mutualisms consist of partners trading services that yield common benefits to both species. Until now, understanding how the payoffs from mutualistic cooperation are allocated among the participants has been problematic. Two hypotheses have been proposed to resolve this problem. The Red Queen effect argues that faster-evolving species are favoured in co-evolutionary processes because they are able to obtain a larger share of benefits. Conversely, the Red King effect argues that the slower-evolving species gains a larger share of benefits. The model we propose shows that the allocations for a common benefit vary when the effect of a reward mechanism is included in the model. The outcome is a shift from the Red Queen effect to the Red King effect and vice versa. In addition, our model shows that either an asymmetry in payoff or an asymmetry in the number of cooperative partners causes a shift between the Red Queen effect and the Red King effect. Even in situations where the evolutionary rates are equal between the two species, asymmetries in rewards and in participant number lead to an uneven allocation of benefits among the partners.

In mutualistic interactions between two species, both species benefit from cooperation. These interactions are ubiquitous in nature. Several well-studied theories have been proposed to explain the evolution and maintenance of mutualisms^1^,^2^,^3^,^4^,^5^. For example, the reciprocity selection theory views the beneficial exchange between species from a market perspective. This theory argues that the cooperative species gain a direct or an indirect benefit. Consequently, choosing a strategy of cooperation increases the fitness of both mutualistic species^6^,^7^. The kin selection theory explains that if, for example, symbiont virulence is transmitted vertically, then related symbionts increase their inclusive fitness by decreasing their total virulence, or even by cooperating with their host, due to the genes they share with other symbiotic individuals^8^,^9^. The authors of the sanction theory argue that hosts display sanctioning behaviours against exploiters or cheaters to maintain the stability of the mutualism^10^,^11^,^12^,^13^. Finally, the predator interference theory considers the case of predation within mutualistic interactions. Predation prevents the population increase of exploiters and favours the maintenance of the mutualism^14^,^15^.

The above theories have focused almost exclusively on how interspecific cooperation can persist over evolutionary time. They have also focused on why mutualistic interactions are conserved, as we would normally expect natural selection to favour exploiters. Cheaters disrupt the mutualistic stability when trying to gain excessive benefits and thereby decrease their contribution to the cooperative relationship^16^. However, although cooperation is maintained between two species by the mechanisms suggested above, how the resulting benefits are allocated to the cooperative species has not yet been extensively studied.

How mutualistic benefits are allocated between interacting species has puzzled empirical scientists for a long time. For example, in mutualistic interactions between a cleaner wrasse *Labroides dimidiatus* and its large client fish, the cleaner wrasse removes ectoparasites on the client fish, taking them as food, and the client fish enjoys a reduced parasite load^17^,^18^. As reported by Bergstrom and Lachmann (2003), in an idealised interaction where there is no potential for cleaners to feed on live tissue or for clients to prey on cleaners^19^, the actual allocation of benefits between a cleaner wrasse and its client fish is relatively straightforward^20^. The allocation of benefits, however, is not as clear in other mutualisms, including ants and lycaenid butterfly caterpillars^21^, plant and pollinators^22^, or symbioses between insects and gut microbes^23^.

To date, two hypotheses have been proposed to address this issue. The Red Queen effect argues that the faster-evolving species should show an advantage in the co-evolutionary process. This effect describes the antagonistic co-evolution between two species over a common ecological resource, for example, between predator and prey or between host and parasite^24^. In 1999, Herre et al. noted that the Red Queen effect can also effectively describe mutualisms because these types of interactions are also an ongoing arms race^4^. By contrast, another school of thought argues that a slower rate of evolution should confer a more favourable outcome when two species compete for the benefits of a mutualism^25^,^26^,^27^. This effect has been called the Red King effect^20^. These two hypotheses are based on the assumption that the cooperative species do not produce any reward, even though such reward mechanisms have been widely observed^28^,^29^,^30^,^31^ (e.g., in the obligate interspecific cooperation between figs and fig wasps, the fig host can reward the cooperative pollinators indirectly by increasing the offspring development ratio^32^,^33^).

The theoretical models of the Red King and Red Queen hypotheses also assume that the distributions of payoffs between species are equal. However, almost all well-documented mutualisms–such as yucca plants and yucca moths^34^,^35^, legumes and nitrogen-fixing bacteria^10^, and figs and fig wasps^33^–have shown that the hosts and symbionts have highly asymmetric payoffs. In principle, the host can therefore always set the rules of the game between mutualistic species. Consistent with this principle, hosts of different mutualisms were recently shown to discriminatively sanction non-cooperative actors and reward cooperative actors to maintain the mutualistic interaction^10^,^13^. Another more extreme phenomenon is when the host completely represses the population of symbionts^36^. Thus, we argue that the reward mechanism and other asymmetric factors (e.g., asymmetric payoffs and asymmetry in the number of players) play a fundamental role in the evolution of mutualisms. In this study, by using a multiplayer game model, we explicitly considered the role of reward mechanisms and asymmetric factors in coevolved mutualisms, and we analysed their effects on the allocation of mutualistic benefits.

## Results

### Model

**Figure 1:**
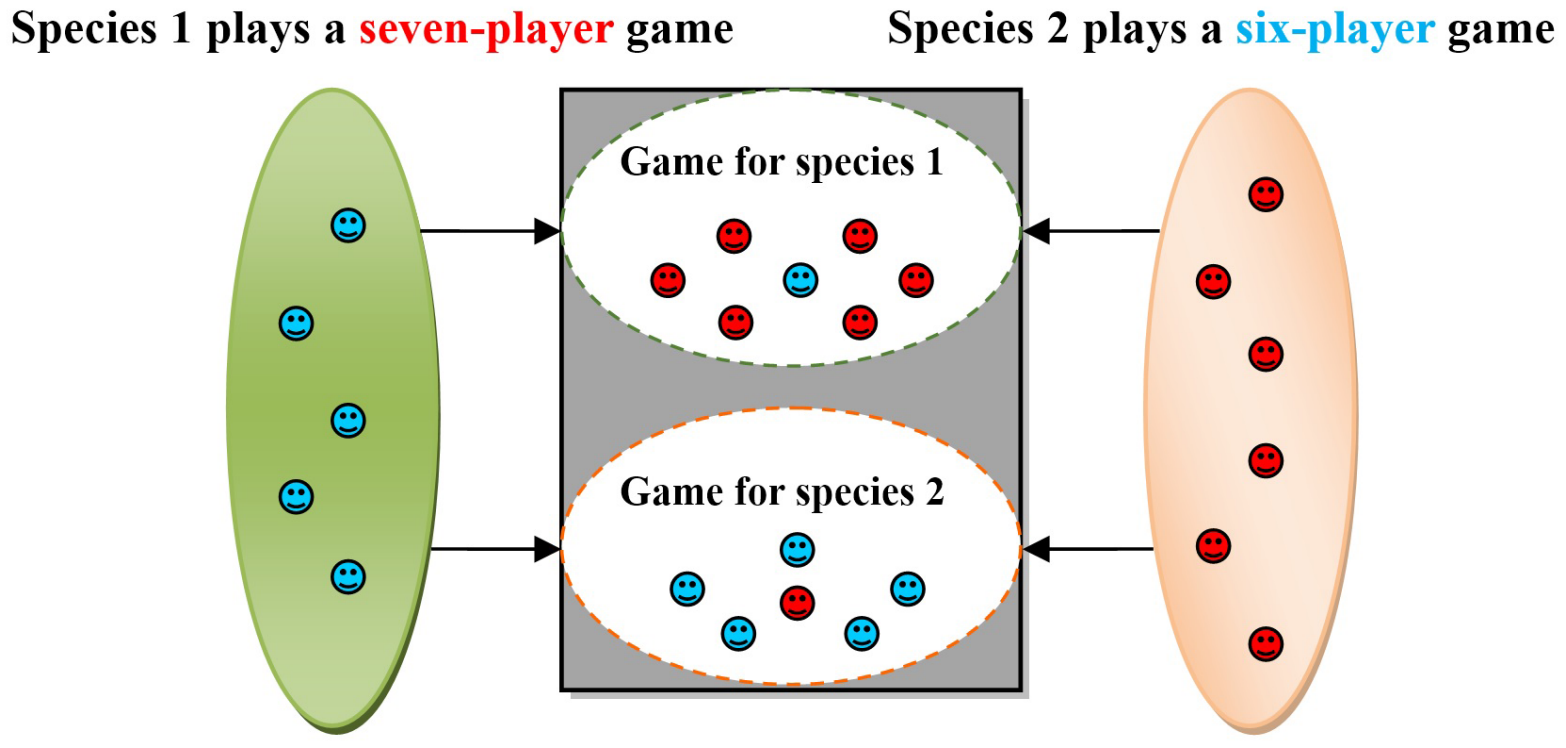
The interactions between species 1 and 2 in mutualisms. We assume that *d*_1_ = 5, *d*_2_ = 6. Species 1 plays in a seven-player game. In this first game, we choose one player of species 1 (the blue faces) to interact with six players of species 2 (the red faces). Species 2 plays a six-player game. In this second game, one player of species 2 joins and interacts with all the players of species 1. Generally, for a *d*_1_ + 1 players' game for species 2, we choose one individual from species 2 to interact with *d*_1_ individuals of species 1. For a *d*_2_ + 1 players' game for species 1, we pick one individual from species 1 to interact with *d*_2_ individuals of species 2. We do not consider intraspecific interactions within groups. It is possible to incorporate even more complexity by considering these interactions within the same species. By doing this, however, the model becomes very convoluted and beyond the scope of this paper. Figure 1 was produced with Microsoft Word. Other figures were obtained with Maple 15.

In this model, we assume that individuals of species 1 and 2 are selected at random from the population and form groups of size *d*_1_ and *d*_2_, respectively. The members of the two species engage in pairwise or multiplayer repeated interactions. The interactions between the two species/groups are shown in Figure 1. At every turn, every individual needs to decide whether to cooperate or to defect. These interactions can thus be depicted by a two-population role-asymmetric game^37^,^38^. The relative payoff matrix is shown in Table 1. In this matrix, the row players belong to one of the species and the column players belong to the other species. For example, a cooperative individual of species 1 obtains a payoff of 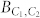 when playing against a cooperative individual of species 2, whereas a cooperative individual of species 2 obtains a payoff of 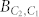. Here, 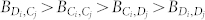 for *i*, *j* = 1, 2 and *i* ≠ *j*. This rank order of payoffs corresponds to the snowdrift game. For a classical, single-species, pairwise snowdrift game, there exists a mixed evolutionary stable state at which the proportion of cooperative individuals is 1 − *c*/(2*b* − *c*). However, for a snowdrift game between two species, this coexistence point is unstable because each species would be better off defecting. Two equilibria arise in which neither player can do better by changing their strategies: one player from species 1 cooperates, and the other from species 2 defects (or vice versa).

The above approach can be extended to multiplayer games. In this case, the payoff we report below (Table 2) is often adopted (reported from Souza et al. 2009)^39^. Here, the cost *c* is shared between cooperative individuals. In addition, it is possible that a particular threshold needs to be met for the mutualistic benefits to materialise. For example, client fishes have been observed to choose cleaning stations with two cleaners over lone cleaners^17^. Similarly, in observations of the interactions between ants and caterpillars, a particular number of ants are required to rescue a caterpillar from its predator. It has also been shown that the quantity of secretions of a lycaenid larva is correlated to the number of attending ants^40^. Generalising from these examples, we assume that the number of cooperative individuals *k* is at least equal to *M* and that the benefit *b* is acquired in turn. For different species, the payoff setup has different values: *b_i_*, *c_i_*, and *M_i_* with 1 ≤ *M_i_* ≤ *d_i_* + 1 and *b_i_* > *c_i_* (*i* = 1,2).

### Multiplayer snowdrift games with reward mechanism and asymmetric factors

The pairwise and multiplayer games considered above do not take into account any reward mechanisms. Such mechanisms, however, have been widely observed in natural mutualistic systems^41^. In addition, previous models may be oversimplified when accounting for these naturally occurring interactions, implying that cooperative individuals interact symmetrically. We argue, instead, that the interactions between cooperative individuals may more often be asymmetric^33^,^38^,^42^,^43^,^44^. To address these limitations, we need a new payoff setup. Another element should also be considered: discrimination between cooperative individuals and defectors is often not possible in many commonly studied mutualisms^45^,^46^.

For these reasons, we use a collective reward mechanism for a multiplayer snowdrift game in a single species, as proposed by Ji et al. (2010)^47^. In their model, the authors allowed cooperative individuals to manifest the fruit of their joint effort earlier on by receiving an additional reward 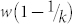. This term gives an estimate of the size of the reward as a function of the number of cooperative individuals *k*^47^. The parameter *w* represents the intensity of the reward. This collective reward usually originates from an external pool of resources (as, e.g., in human societies^48^), or it might take the form of synergistic effects in some biological systems (where the benefits will increase with the increasing number of cooperative individuals)^49^,^50^. By definition, a collective reward is imposed on all players either because there is no way to detect the behaviour of individual partners (or its effects) or because the differences between cooperative and non-cooperative individuals are too small to be detected (as in the legume–rhizobium mutualism^51^). Under these assumptions, we can obtain the payoffs values for a cooperative individual and a defector of species *i* as: 
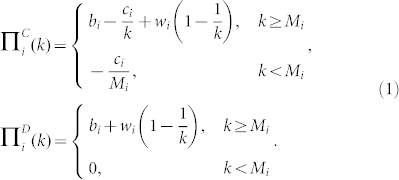
where the parameters *b_i_, c_i_, M_i_* and *w_i_* are different for species 1 and 2.

### Local dynamics of mutualism

In our model, we study cooperative behaviour in an infinite, well-mixed population. It is also our intent to compute the fractions of cooperative/non-cooperative individuals in the population. The cooperation frequencies of the players of species 1 and species 2 are *x*(*t*) and *y*(*t*), respectively. The fitness functions of a cooperative individual of species 1 and species 2 are 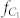 and 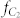, respectively. Based on the interactions occurring between the species, the fitness 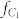 depends on the frequency *y* of cooperative players of species 2, 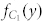. Similarly, the fitness 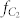 depends on the frequency *x* of cooperative players of species 1, 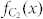. By randomly sampling the groups^39^,^52^, we obtained the following average fitness of a cooperative individual and a defector of species 1 and 2: 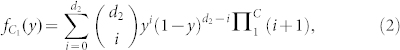

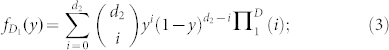
and 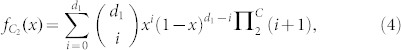

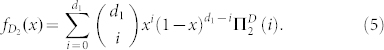


Thus, the average payoffs for a player of species 1 and 2 are 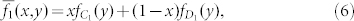

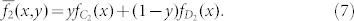


The replicator dynamics assume that the per capita growth rate in the population is determined by the difference between the payoff for a particular strategy and the average payoff 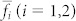
53,54,55. Thus, the evolutionary times of *x*(*t*) and *y*(*t*) are governed by 
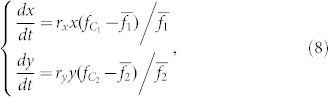
where *r_x_* and *r_y_* are the evolutionary rates of the two species.

**Figure 2:**
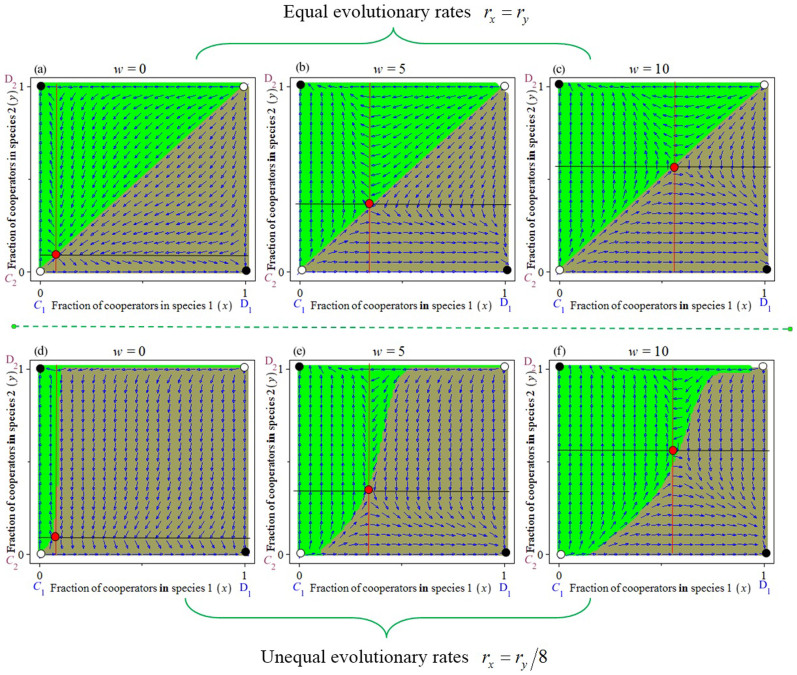
The shift between the Red Queen and the Red King effect, accounting for reward mechanisms. The black solid circles are locally stable strategies (ESSs), and the empty circles are unstable strategies (non-ESSs). The red solid circles denote inner equilibrium points that are unstable. For equal evolutionary rates, *r_x_* = *r_y_*, the basins of attraction of the two equilibriums (*C*_1_, *D*_2_) and (*D*_1_, *C*_2_) are of equal size ((a), (b) and (c)), whereas the size of these two basins is unequal when the evolutionary rates between two species are asymmetric ((d), (e) and (f)). The colours illustrate the regions leading to the equilibria favourable to species 1 (green region leading to (*D*_1_, *C*_2_)) and species 2 (grey region leading to (*C*_1_, *D*_2_)). For a multiplayer game without a reward mechanism (with *i* = 1,2 and *d_i_* = 19, *w_i_* = 0), the basin of attraction favourable to faster-evolving species grows substantially. However, the internal equilibrium points shift with an increase in the collective reward (with *i* = 1,2 and *d_i_* = 19, *w_i_* = 5), as in (e). As a result, the Red King effect eventually appears with increasing intensity of the reward, as in (f). This causes a switch from the Red Queen effect (d) to Red King effect (f). The evolutionary rates are *r_x_* = 1/8 and *r_y_* = 1 in (d), (e), (f). The other parameters are fixed at *b_i_* = 2, *c_i_* = 1 and *M_i_* = 1.

We analysed the dynamics of this game using nonlinear dynamic theory^56^ and simulations (see the Methods section for more details). Two locally stable equilibrium points (*C*_1_, *D*_2_) and (*D*_1_, *C*_2_) arise in our model if the reward *w_i_* satisfies *w_i_*/*b_i_* < *d_j_c_i_*/*b_i_* (with *i*, *j* = 1,2 and *i* ≠ *j*). The pair of equilibrium points (*x*,*y*) in each cell corresponds to the frequency of cooperation of species 1 and 2, respectively. In addition, the equilibrium points (*C*_1_, *D*_2_) and (*D*_1_, *C*_2_) are denoted by *E*_2_(1,0) and *E*_3_(0,1). Thus, the equilibrium points (*C*_1_, *D*_2_) and (*D*_1_, *C*_2_) occur where the players of one species are all cooperating and the players of the other species are all defecting. It is important to note that these two stable points have their own basins of attraction (obtaining these basins of attraction has proven extremely useful for analysis of such behaviours^25^,^57^,^58^). As we show in Fig. 2, the ‘grey region’ and the ‘green region’ are the basins of attraction for (*C*_1_, *D*_2_) and (*D*_1_, *C*_2_), respectively. In effect, the solution trajectories converge to the point (*C*_1_, *D*_2_) if the initial strategy frequencies of each species fall within the ‘grey region’, whereas they converge to the point (*D*_1_, *C*_2_) if the initial strategy frequencies fall within the ‘green region’ (see Fig. 2).

We propose a method to calculate the benefits to the players of the two species when the stability of mutualisms can be maintained. We begin by assuming that the threshold is *M_i_* = 1 (with *i* = 1,2) and that the number of players is *d*_1_ = *d*_2_ for species 1 and 2. The size of the ‘grey region’ and the ‘green region’ is defined as *S*_1_ and *S*_2_, respectively (0 < *S*_1_, *S*_2_ < 1, *S*_1_ + *S*_2_ = 1). The players of cooperative games will eventually adopt an ESS of (*C*_1_, *D*_2_) with probability *S*_1_ and an ESS of (*D*_1_, *C*_2_) with probability *S*_2_. The fitness of the players of species 1 and 2 are thus denoted by *B*_1_ = *S*_1_·(*b*_1_ − *c*_1_) + *S*_2_·*b*_1_ and *B*_2_ = *S*_1_·*b*_2_ + *S*_2_·(*b*_2_ − *c*_2_), respectively. As a result, species 1 will obtain a larger share of returns than species 2 when *B*_1_ > *B*_2_, and species 2 will obtain a larger share of returns than species 1 when *B*_1_ < *B*_2_. In addition, the two species obtain equal benefits when *B*_1_ = *B*_2 _(for more details, see the Supplementary Information section). This implies that, in mutualisms, the division of the benefits (surplus) depends on the size of the basins, *S_i_*; on the benefits, *b_i_*; and on the costs, *c_i_* (with *i* = 1,2). Using economics terminology, mutualistic benefits can be named as a ‘surplus’ generated by the mutualism^20^.

When the benefit *b_i_*and the cost *c_i_*of the two species are the same, we return to the methods used in previous studies^25^,^57^. Under these conditions, the allocations of mutualistic benefits appear to rely on the size of the respective basins of attraction. In other words, species 1 will reap larger benefits than species 2 when the basin of attraction of (*D*_1_, *C*_2_) is larger than (*C*_1_, *D*_2_), whereas species 2 will reap larger benefits than species 1 when the basin of attraction of (*C*_1_, *D*_2_) is larger than (*D*_1_, *C*_2_). Intuitively, the size of the basins might reflect the behaviours/strategies of the individuals involved (e.g., the resilience or the lifetime of the state). These behaviours should result from having to adapt to a changing environment over evolutionary time. For each individual in species 1 and 2, the size of the basins of attraction reflects the probability of defection. Individuals with a higher probability of defection will reap larger benefits. In addition, for each species, the size of the basins of attraction reflects the proportion of defectors. The higher the proportion of defectors, the greater the benefit for that species.

## Simulations and Discussion

### The allocation of mutualistic benefits under asymmetric evolutionary rates

#### Reward effect

**Figure 3:**
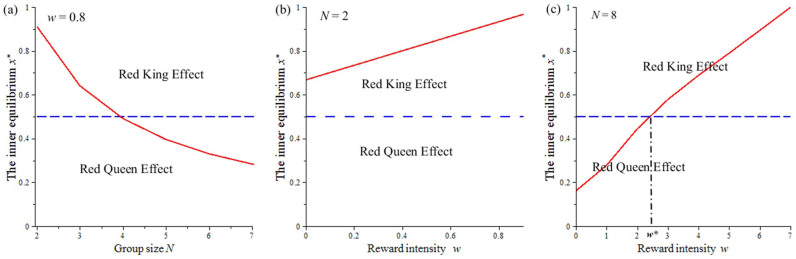
The effects of a reward *w_i_* and the game size *N* on the internal equilibrium x*. We assume the numbers of players between species to be equal, i.e., *d*_1_ = *d*_2_ = *d*. Thus, the game size for each species is defined by *N*, and *N* = *d* + 1. (a) Given a relatively low reward intensity (i.e., *w* = 0.8), the internal equilibrium x* is above 0.5 in small groups (e.g., *N* = 2 or *N* = 3). (b) For pairwise games, the equilibrium is always above 0.5 for any reward intensity. (c) When the size of the mutualistic group is large (i.e., *N* = 8), the internal equilibrium can shift from a small value (below 0.5) to a large value (above 0.5) with a rise in the collective reward. The other parameters are fixed at *b_i_* = 2, *c_i_* = 1 and *M_i_* = 1.

In this section, we assume that the benefit *b_i_*, the cost *c_i_*, the number of players *d_i_*, and the reward *w_i_* of the two species are the same. If the evolutionary rates are unequal (i.e., species 1 with rate *r_x_* = *r_y_*/8), the basins of attraction of (*C*_1_, *D*_2_) and (*D*_1_, *C*_2_) become unequal in size. In other words, the allocation of the resulting benefits is unequal between the two species (Fig. 2d, 2e, and 2f). If the game is run without any reward mechanism (with *w_i_* = 0), the basin of attraction (*C*_1_, *D*_2_) is larger than the basin of attraction (*D*_1_, *C*_2_). This implies that the faster-evolving species will receive a larger share of the benefits (Fig. 2d). This is in line with what is predicted by the Red Queen effect^24^. However, this effect can shift to the Red King effect when we include the reward variable in the multiplayer game. Specifically, the size of the basin of attraction of (*D*_1_, *C*_2_) increases substantially as the reward intensity *w_i_* increases (Fig. 2e). As a result, the slower-evolving species obtains a larger share of the benefits (Fig. 2f). This means that the initial Red Queen effect shifts to the Red King effect when the magnitude of the reward is over a certain threshold value (Fig. 3c). However, this reward mechanism does not allow the Red King effect to shift to the Red Queen effect (Fig. 3a and 3b). Finally, we find that the size of the reward can accentuate the Red King effect (Fig. 3b and 3c).

Next, we explore the process resulting from the shift between the Red Queen effect and the Red King effect. From the analyses above, we predict which species would be favoured. As explained, this depends on the relative sizes of the two basins of attraction (*C*_1_, *D*_2_) and (*D*_1_, *C*_2_), i.e., *S*_1_ and *S*_2_. Theoretically, it is difficult to obtain accurate values for *S*_1_ and *S*_2_. Fortunately, when the evolutionary game is symmetric, we can use values of *x* and *y* to measure the size of *S*_1_ and *S*_2 _(in this case, *x* = *y*; further details of this method are reported by Bergstrom and Lachmann (2003)^25^). Specifically, the replicator solutions of the two species create quadrants in the state space (0 ≤ *x*, *y* ≤ 1) (Fig. 2). When the internal equilibrium is *x* = *y* = 0.5, the bottom-left and the top-right quadrants are of equal size. As a result of this symmetric geometry, the basins of attraction (*C*_1_, *D*_2_) and (*D*_1_, *C*_2_) become the same, i.e., *S*_1_ = *S*_2_. If the equilibrium is less than *x* = 0.5, the bottom-left quadrant is smaller than the top-right quadrant, i.e., *S*_1_ > *S*_2_ (Fig. 2d). As a result, the faster-evolving species would be favoured, as depicted by the Red Queen effect (Fig. 3c). Conversely, if the equilibrium is greater than *x* = 0.5, the bottom-left quadrant is larger than the top-right quadrant, i.e., *S*_1_ < *S*_2_ (Fig. 2f). Thus, the evolutionary process will favour the slower-evolving species, as described by the Red King effect^25^ (Fig. 3c). Interestingly, in a multiplayer game with fixed *b_i_*, *c_i_*, and a fixed number of players, *d_i_*_,_ the equilibrium *x* exceeds 0.5 with increasing rewards (Fig. 3c). Thus, the Red Queen effect can shift to the Red King effect and vice versa, depending on the intensity of the rewards. Finally, note that this method is not valid when the evolutionary game is asymmetric (e.g., with asymmetric payoffs or asymmetries in the number of players) because the above geometry becomes asymmetric (with *x* ≠ *y*, see Fig. S9 in the electronic Supplementary Material). In this case, we use simulations to estimate the size of *S*_1_ and *S*_2_.

#### Asymmetric players

**Figure 4:**
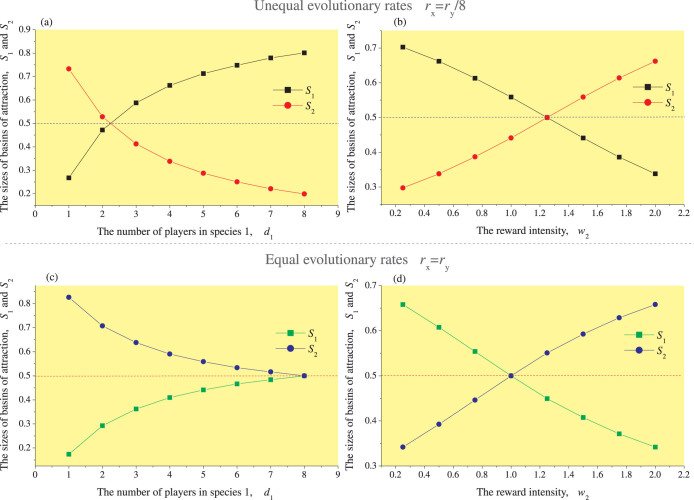
The influence of asymmetric players or rewards on the allocation of mutualistic benefits. (1) First, we consider unequal evolutionary rates between the two mutualistic species (*r_x_* = 1/8, *r_y_* = 1). (a) For a fixed number of players of species 2 (*d*_2_ = 8), the difference between *S*_1_ and *S*_2_ depends on the number of players of species 1 (*d*_1_), where *S*_1_ and *S*_2_ represent the size of the basins of attraction of the equilibrium (*C*_1_, *D*_2_) and (*D*_1_, *C*_2_), respectively. For one-to-many interactions between the two species, *S*_2_ is larger than *S*_1_. Conversely, *S*_1_ is larger than *S*_2_ when the interactions between species are many-to-many (e.g., *d*_1_ = 4). (b) Similarly, by adjusting the intensity of reward for species 2 (*w*_2_), the Red King effect (i.e., *S*_2_ > *S*_1_) can also shift into the Red Queen effect (i.e., *S*_1_ > *S*_2_), and vice versa (the reward intensity on species 1 is fixed at *w*_1_ = 1). (2) For equal evolutionary rates between two species (*r_x_* = *r_y_* = 1), each player will receive equal mutualistic benefits when the number of players of the two species are symmetric (c) (e.g., *d_i_* = 8 with *i* = 1,2). When the numbers of the players of the two species are asymmetric (*d*_1_ ≠ 8), each player will receive unequal mutualistic benefits. Similar results can be obtained for the rewards (as in (d)). We also assume that *d*_2_ = 8 in (c) and *w*_1_ = 1 in (d). The other parameters are fixed at *b_i_* = 2, *c_i_* = 1 and *M_i_* = 1, *w*_1_ = *w*_2_ = 0 in (a) and (c), and *d*_1_ = *d*_2_ = 4 in (b) and (d).

Surprisingly, only recently there has been some focus on the effect of asymmetry in the number of interacting partners (i.e. *d*_1_ ≠ *d*_2_)^33^,^57^. We know that this phenomenon is ubiquitous in nature. Most literature on mutualisms assumes that a single host interacts with, and controls the fate of, multiple partners. In the fig and fig wasps mutualism, for example, a single fig is attended by multiple fig wasps. From the perspective of each wasp, this is a pairwise game, but from the perspective of the fig, this is a multiplayer game. However, for symbioses between plants and arbuscular mycorrhizal fungi, plant species are typically colonised by multiple fungal species^59^, and fungal individuals can simultaneously interact with multiple host plants^60^ or species^61^. For one-to-many interactions, when a symbiont evolves much faster than its host–which is often the case–the host can dominate the co-evolutionary process by allowing the Red King effect to appear (see Fig. 4a or Fig. S9a). By contrast, the symbiont becomes dominant in the co-evolutionary race when we consider many-to-many interactions (e.g., the interaction between plants and arbuscular mycorrhizal fungi) (see Fig. 4a or Fig. S9b). This is consistent with the more often cited Red Queen effect. Thus, we conclude that asymmetry in the number of players can produce different effects (i.e., the Red Queen versus Red King). This result is consistent with those of Gokhale and Traulsen (2012).

#### Asymmetric payoffs

In mutualistic interactions, the interacting players can obtain different rewards at different times (i.e.*w*_1_ ≠ *w*_2_). The Red King effect can shift to the Red Queen effect by adjusting reward asymmetry. Similarly, the Red Queen effect might be reversed by adjusting reward asymmetry (Fig. 4b or Fig. S9c and 9d). In fact, the payoff matrices become asymmetric because of the different rewards *w_i_*, benefits *b_i_* and costs *c_i_* for the two species.

In summary, the reward mechanism and the asymmetry of interactions (e.g., asymmetry in payoffs or the number of players) might determine the ultimate allocation of mutualistic benefits (the Red Queen effect or the Red King effect). These results might provide an explanation for why, in some mutualisms, the faster-evolving species are dominant (e.g., in the endophytic fungi and grasses mutualism, the fungi appear to reduce the host's tendency to reproduce sexually; sex seems to be the critical element^4^). In other mutualisms, the slower-evolving species are dominant (e.g., in the invertebrate-algal and lichen mutualisms^62^,^63^ or the host-endosymbiont mutualisms^64^). Therefore, opposite outcomes may result from differences in the payoffs, in the number of players, and in the reward mechanism across species.

### The allocation of mutualistic benefits under equal evolutionary rates

Given symmetrically interacting species with equal evolutionary rates, the allocation of benefits between the two species will be equal (Fig. 4c or Fig. S9e). In the case of asymmetry, however, the species with fewer participants will receive relatively more benefits (Fig. 4c or Fig. S9f). In addition, the asymmetric rewards imply that benefits are allocated unevenly. If the rewards are equal, the benefits should also be equal (Fig. 4d or Fig. S9g). Instead, the species that gets fewer rewards will have a stronger tendency to freeload and will receive greater benefits without paying the related costs (Fig. 4d or Fig. S9h). In addition, we can assume that the asymmetry in initial benefits or costs causes an unequal allocation of resulting benefits.

Note that these specific results are suitable for the allocation of benefits within a single species constituted by two types of players with equal evolutionary rates. In specific instances, the resulting benefits arising from the cooperative behaviour may be allocated to the participants according to a set of rules (e.g., a social hierarchy). This often happens in social animal groups, e.g., in lions and wolves when displaying cooperative hunting behaviour.

### Complex Cooperation

**Figure 5:**
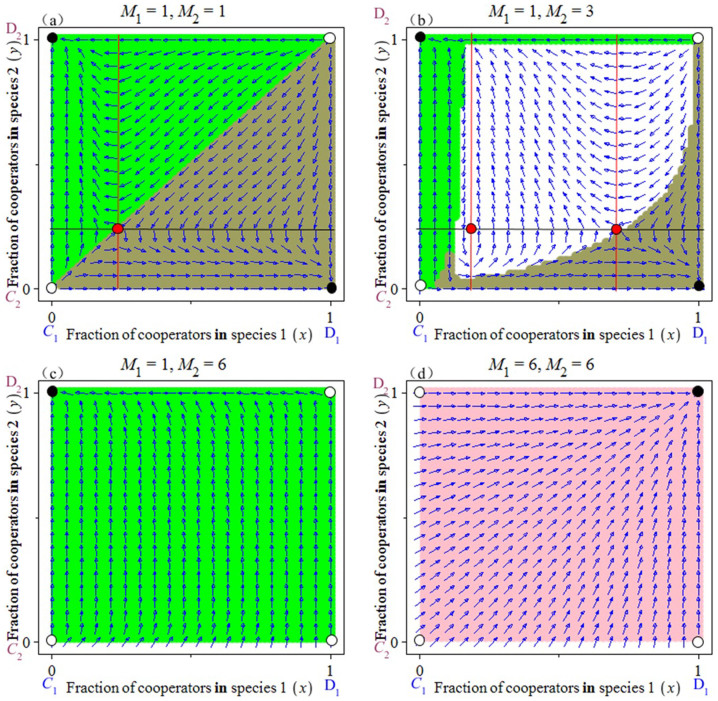
Complex cooperative behaviours in the snowdrift game with a reward or a general threshold. (a) If the thresholds for the two species are *M*_1_ = 1 and *M*_2_ = 1, a bistability is observed between all cooperative individuals and all defectors. (b) When *M*_1_ = 1, *M*_2_ = 3, we observe the “white regions” with closed orbits in the interior. The initial conditions falling within the “grey region” will converge to the equilibrium (*C*_1_, *D*_2_) and those falling within the “green region” will converge to the equilibrium (*D*_1_, *C*_2_). (c) When *M*_1_ = 1, *M*_2_ = 6, all initial conditions lead to (*D*_1_, *C*_2_). At this point, only one side chooses to cooperate while the other side chooses to defect. (d) In the special case of *M_i_* = *d_i_*+1 and *w_i_* = 0 with *i* = 1,2, the equilibrium point (*C*_1_, *C*_2_) (the ‘pink region’) is an ESS. In this case, full cooperation among players becomes possible. The other parameters are fixed at *d_i_* = 4, *b_i_* = 2, *c_i_* = 1,*w_i_* = 0 with *i* = 1,2, and *r_x_* = *r_y_* = 1.

Different social dilemmas can arise within the context of multiplayer games. When the thresholds *M_i_* are adjusted, we incur a scenario of complex cooperation. In adjusting the thresholds *M_i_*between two species, not only do we observe a bistability between all cooperative individuals and all defectors (Fig. 5a) but we also find that the cooperative individuals and the defectors can coexist through a rock-paper-scissors dynamic (as shown by the “white region” with closed orbits in Fig. 5b). Furthermore, by accounting for an asymmetry in the thresholds (i.e., *M*_1_ ≠ *M*_2_), the cooperative system follows the boxed pigs game, in which only one side chooses to cooperate, but the other side inevitably defects (Fig. 5c).

Finally, yet importantly, a special case is represented by those human societies showing a division of labour. For a functioning cooperation, it is required that each player cooperates to complete the task (*M_i_* = *d_i_* + 1). In such situations, we witness an extreme outcome: a stable state of full cooperation arises (i.e., *E*_4_(1,1) is an ESS – the ‘pink region’ in Fig. 5d).

## Conclusion

To analyse how benefits are shared between two mutualistic species, we established a multiplayer snowdrift game model that incorporated a reward mechanism. This model demonstrates that the Red Queen effect can shift to the Red King effect and vice versa by adjusting the intensity of the rewards. When the rewards are small, the Red Queen effect is observed; when the rewards exceed a certain threshold, the Red Queen effect transforms into the Red King effect. To make the model more realistic, we also included asymmetric payoffs and asymmetries in the number of interacting partners. By changing the degree of asymmetry of the reward or the number of interacting partners, we again found that the Red Queen effect can shift to the Red King effect. Even in situations where the evolutionary rates are equal between the two species, the asymmetric factors (e.g., asymmetry in the reward *w_i_*, in the benefits *b_i_*, in the costs *c_i_* or in the different number of interacting individuals belonging to the two species *d_i_*) can create an inequality in the allocation of the resulting benefits between the partners.

Moreover, some complex cooperative strategies might be observed by manipulating the thresholds *M_i_*. First, for more general thresholds (i.e., with *M_i_* > 1 and *i* = 1,2), the cooperative individuals and the defectors seem to coexist with oscillating frequencies. Second, when there is an asymmetry in the thresholds (i.e., *M*_1_ ≠ *M*_2_), the system falls into the dilemma of the boxed pigs game, in which only one side chooses to cooperate and the other side chooses to defect. Third, in the special case of all participants cooperating, a benefit is generated such that a state of full cooperation becomes stable in the population.

## Methods

### The dynamics of an evolutionary snowdrift game with a reward mechanism

The evolutionary times of *x*(*t*) and *y*(*t*) of the two species are governed by the following replicator dynamic equations^25^,^57^

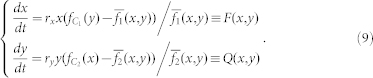


From equation (9), we can obtain four boundary equilibrium points denoted as *E*_1_(0,0), *E*_2_(1,0), *E*_3_(0,1) and *E*_4_(1,1). In addition, using simulations, we can obtain the inner equilibrium points and denote them as: *E*(*x_i_*,*y_i_*) where *i* = 1,2. The elements of vector (*x*,*y*) are the frequencies of the cooperative individuals of species 1 and 2, respectively. It is, however, difficult to obtain the analytical expressions of *x_i_*and *y_i_* for large group sizes *N* because the inner equilibrium points should satisfy the following equations with *N* powers 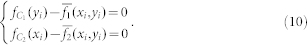


The stability of the nonlinear system can be determined from the analysis of linearisation^56^. Thus, to analyse the evolutionary stability of the replicator dynamics (9) with *N* *+* *1* powers, we should first obtain the linearisation of the dynamics at every equilibrium point. The linearisation of the replicator dynamics (9) at an equilibrium point 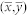 becomes 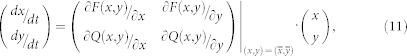
where 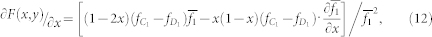

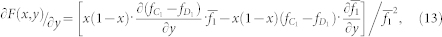

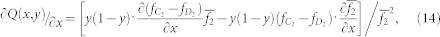
and 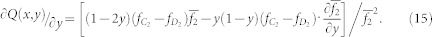


To simplify this analysis, we assume that the benefits *b_i_*, the costs *c_i_*, the rewards *w_i_*, and the thresholds *M_i_*(with *i* = 1,2) of the two species are the same. By analysing the property of the matrices' eigenvalues, produced by the linearisation of the replicator dynamics (11) at the equilibrium points, we find that:

Scenario (1) 1 ≤ *M_i_* < *d_i_* + 1 with *i* = 1,2. The equilibrium points *E*_1_(0,0), *E*(*x*_1_,*y*_1_) and *E*(*x*_2_,*y*_2_) are sources (unstable); the equilibrium points *E*_2_(1,0) and *E*_3_(0,1) are locally stable, if *w_i_*/*b_i_* < *d_j_c_i_*/*b_i_*.with *i*,*j* = 1,2 and *i* ≠ *j*. In addition, the equilibrium point *E*_4_(1,1) is a sink (stable), if *w_i_*/*b_i_* > *d_j_c_i_*/*b_i_*;

Scenario (2) *M_i_* = *d_i_* + 1 with *i* = 1,2. The equilibrium points *E*_1_(0,0), *E*_2_(1,0) and *E*_3_(0,1) are sources (unstable), and the equilibrium point *E*_4_(1,1) is a sink (stable).

## Author Contributions

Conceived and designed the model: R.W.W. and Y.T.L. Performed the research: L.G. Analysed the model: L.G. Wrote the paper: R.W.W. and L.G. All authors revised the manuscript and approved the final version.

## Supplementary Material

Supplementary Information Electrical Supplementary Information

## Figures and Tables

**Table 1:** Payoff matrix of the pairwise snowdrift game between two species

		Species 2				Species 1	
		*C*_2_	*D*_2_			*C*_1_	*D*_1_
Species 1	*C*_1_	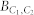	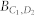	Species 2	*C*_2_	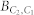	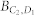
	*D*_1_	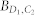	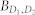		*D*_2_	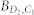	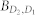

**Table 2:** Payoff values of the multiplayer snowdrift game

Payoff obtained	Cooperation	Defection
1 ≤ k < M	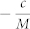	0
k ≥ M	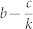	*b*
