# Supplementary material for: The shift between the Red Queen and the Red King effects in mutualisms
Source: Sci Rep. 2015 Feb 4;5:8237. doi: 10.1038/srep08237 (PMC4316171; doi:10.1038/srep08237)
Supplement: Supplementary Information — Electrical Supplementary Information [file srep08237-s1.pdf]

# Supplementary Information

## The shift between the Red Queen and the Red King effects in mutualisms

*Authors*: Lei Gao[1,2], Yao-Tang Li[2,*] & Rui-Wu Wang[1,*]

[1]School of Mathematics and Statistics, Yunnan University, Kunming, Yunnan, 650091, P.R. China.

[2]State Key Laboratory of Genetic Resources and Evolution, Kunming Institute of Zoology, Chinese Academy of Science, Kunming, Yunnan, 650223, P.R. China.

*Corresponding author: Rui-Wu Wang; Yao-Tang Li;

E-mails: wangrw@mail.kiz.ac.cn; liyaotang@ynu.edu.cn.

**Payoffs to Players and the Reward Function in Multiplayer Snowdrift Games**

The pairwise snowdrift game imagines the situation of two drivers stuck at a snowdrift blocking their way home. The cost they incur to remove the snowdrift is $c$. By getting home, they obtain benefit $b$. The two drivers can decide either to cooperate (to shovel) or to defect (not to shovel). Shovelling together yields a benefit $b$ to both players. The cost $c$ of shovelling is shared when both cooperate (see the payoff matrix in Table S3).

When we consider $d$ players in this game, the payoffs for a cooperative individual and a defector become[1]

$$\prod_C(k)=\begin{cases} b-\dfrac{c}{k}, & k\geq M \\ -\dfrac{c}{M}, & k<M \end{cases}, \quad \prod_D(k)=\begin{cases} b, & k\geq M \\ 0, & k<M \end{cases}, \tag{S1}$$

where $k$ is the number of cooperative individuals in the group. A benefit is produced only if there are at least $M$ ($1 \le M \le d$) cooperative individuals.

By resorting to the collective reward mechanism, as proposed by Ji et al. (2010)[2], we can give the payoffs for a cooperative individual and a defector of species $i$ (with $i = 1, 2$), as

$$\prod_i^C(k) = \begin{cases} b_i - \dfrac{c_i}{k} + w_i\left(1 - \dfrac{1}{k}\right), & k \ge M_i \\ -\dfrac{c_i}{M_i}, & k < M_i \end{cases}, \quad \prod_i^D(k) = \begin{cases} b_i + w_i\left(1 - \dfrac{1}{k}\right), & k \ge M_i \\ 0, & k < M_i \end{cases}, \quad \text{(S2)}$$

where $w_i\left(1 - {}^{1}\!/_{k}\right)$ is the additional reward of completing the work sooner, when more cooperative individuals share the shovelling. Through their joint effort, all players receive an additional reward of $w_i\left(1 - {}^{1}\!/_{k}\right)$. This reward is a function of the number of cooperative individuals $k$. The parameter $w_i$ represents the intensity of rewards.

Here, the size of the reward (denoted by $R(k) = w_i\left(1 - {}^{1}\!/_{k}\right)$) increases nonlinearly with the number of cooperative individuals $k$, and it satisfies the economic law of "diminishing the marginal utility"[3] (i.e., the rate of the change of the rewards decreases with the increasing number of cooperative individuals, and it is approaches 0 if $k \to \infty$, i.e., $\lim_{k \to \infty} \dfrac{dR(k)}{dk} = 0$). In addition, we note that $R(k)$ reaches its maximum under these assumptions. In this case, it is denoted as $R_{\max} = w_i$ (see Fig. S7). Similarly, the payoff function (i.e., $\prod_i^C(k)$ and $\prod_i^D(k)$) is a saturation (nonlinear) function of the number of cooperative individuals $k$ (see Fig. S8).

**The Fitness of the Two Species**

In Fig. 1, the 'grey regions' and the 'green regions' are the basins of attraction of the equilibrium points $E_2(1,0)$ and $E_3(0,1)$, respectively. The size of the 'grey regions' and the 'green regions' are defined as $S_1$ and $S_2$ $(0 < S_1,\ S_2 < 1, S_1 + S_2 = 1)$. The participants of the cooperative games will eventually adopt an ESS of $E_2(1,0)$ with probability $S_1$, and adopt an ESS of $E_3(0,1)$ with probability $S_2$. The fitness of the players of species 1 and 2 are thus given by

$$B_1 = S_1 \cdot \overline{f_1}\Big|_{E_2(1,0)} + S_2 \cdot \overline{f_1}\Big|_{E_3(0,1)} = S_1 \cdot \left(b_1 - c_1\right) + S_2 \cdot b_1 = b_1 - S_1 \cdot c_1, \tag{S3}$$

$$B_2 = S_1 \cdot \overline{f_2}\Big|_{E_2(1,0)} + S_2 \cdot \overline{f_2}\Big|_{E_3(0,1)} = S_1 \cdot b_2 + S_2 \cdot \left(b_2 - c_2\right) = b_2 - S_2 \cdot c_2. \tag{S4}$$

where $S_1 \cdot \overline{f_1}\Big|_{E_2(1,0)}$ and $S_2 \cdot \overline{f_1}\Big|_{E_3(0,1)}$ are the fitness of players of species 1 who adopt the strategies $E_2(1,0)$ and $E_3(0,1)$. $S_1 \cdot \overline{f_2}\Big|_{E_2(1,0)}$ and $S_2 \cdot \overline{f_2}\Big|_{E_3(0,1)}$ are the fitness of players of species 2 who adopt strategies $E_2(1,0)$ and $E_3(0,1)$. Based on these assumptions, we can obtain the following results:

(i) When $B_1 > B_2$, species 1 will receive a larger share of benefits than species 2;

(ii) When $B_1 < B_2$, species 2 will receive a larger share of benefits than species 1;

(iii) When $B_1 = B_2$, the two species will receive equal benefits.

## References


1 Souza, M. O., Pacheco, J. M. & Santos, F. C. Evolution of cooperation under N-person snowdrift games. *J. Theor. Biol.* **260**, 581-588 (2009).

2 Ji, M., Xu, C., Zheng, D.-F. & Hui, P. Enhanced cooperation and harmonious population in an evolutionary N-person snowdrift game. *Physica A* **389**, 1071-1076 (2010).

3 R McConnell, C. Economics Principles, Problems, and Policies (2008).

**Table Legend**

**Table S3: Payoff matrix of the pairwise snowdrift game**

| | | *Player* 2 | |
|---|---|---|---|
| | | *C* | *D* |
| *Player* 1 | *C* | $b-\frac{c}{2}$ | $b-c$ |
| | *D* | $b$ | $0$ |

## Figure Legends

**Figure S7. The effects of the number of cooperators on the amount of the reward.** The reward function is $w_i\left(1-1/k\right)$. To simplify, we assume the intensity of the rewards between the species to be equal, i.e. $w_1 = w_2 = w$ . The other parameters are fixed at $b_1 = b_2 = 2,\ c_1 = c_2 = 1$ and $M_1 = M_2 = 1$.

**Figure S8. The effects of the number of cooperators on the payoff of a cooperator/defector.** We also assume $w_1 = w_2 = w$ for both species 1 and 2. The other parameters are fixed at $b_1 = b_2 = 2,\ c_1 = c_2 = 1$ and $M_1 = M_2 = 1$.

**Figure S9. The influence of asymmetric players or rewards on the allocation of mutualistic benefits.** (1) First, we consider the evolutionary rates to be different between the two mutualistic species ( $r_x = 1/8, r_y = 1$). (a) For one-to-many interactions between two species, the basin of attraction of the equilibrium $\left(D_1, C_2\right)$ is larger than the one of the equilibrium $\left(C_1, D_2\right)$. Conversely, when the interaction between the species is many-to-many, the basin of attraction of the equilibrium $\left(C_1, D_2\right)$ is larger than the one of the equilibrium $\left(D_1, C_2\right)$ (b). The Red King effect appears when the reward for each mutualistic species is symmetric (c) ( $w_1 = 2, w_2 = 2$ ). The Red Queen effect appears instead when the rewards are asymmetric (d) ( $w_1 = 2, w_2 = 1$). (2) For equal evolutionary rates between two species ( $r_x = r_y = 1$ ), each player obtains equal mutualistic benefits when the number of players of the two species are symmetric (e) ( $d_i = 2$ with $i = 1, 2$ ). Each player obtains unequal mutualistic benefits, however, when the numbers of the players of the two species are asymmetric (f) ( $d_1 = 1,\ d_2 = 2$ ). Similar results can be obtained for the rewards (as in (g) and (h)). The other parameters are fixed at $b_i = 2,\ c_i = 1$ and $M_i = 1$; $w_i = 0$ in (a), (b), (e) and (f); $d_1 = d_2 = 4$ in (c), (d), (g) and (h).

**Figure S7.**

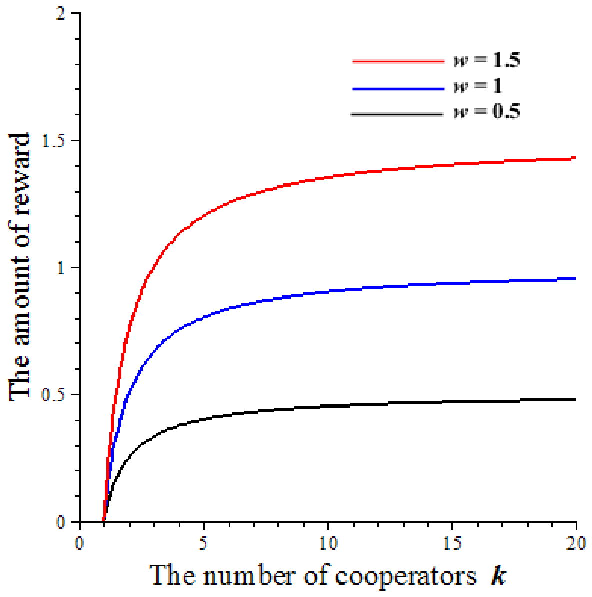
w = 1.5
w = 1
w = 0.5
The amount of reward
The number of cooperators k
0
0.5
1
1.5
2
0
5
10
15
20

**Figure S8.**

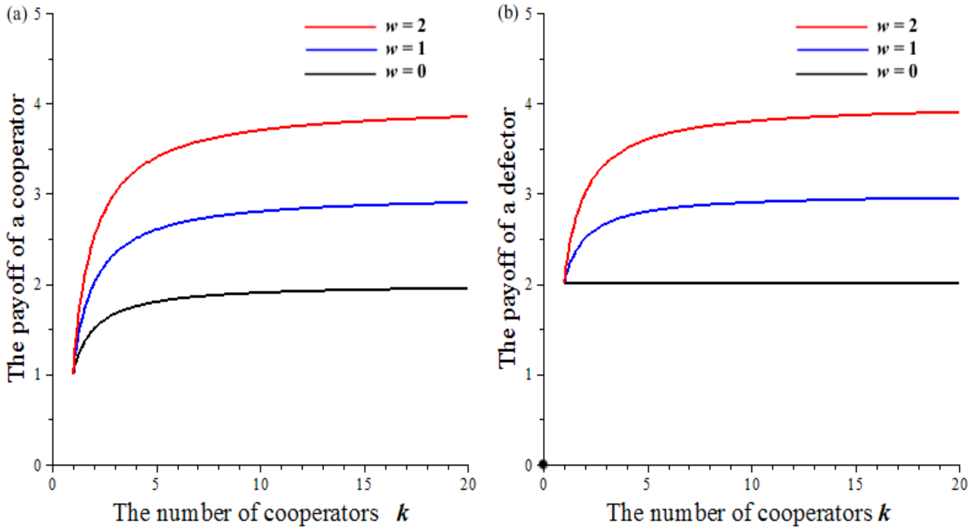

(a)
The payoff of a cooperator
The number of cooperators $k$
$w = 2$
$w = 1$
$w = 0$
(b)
The payoff of a defector
The number of cooperators $k$
$w = 2$
$w = 1$
$w = 0$

**Figure S9.**

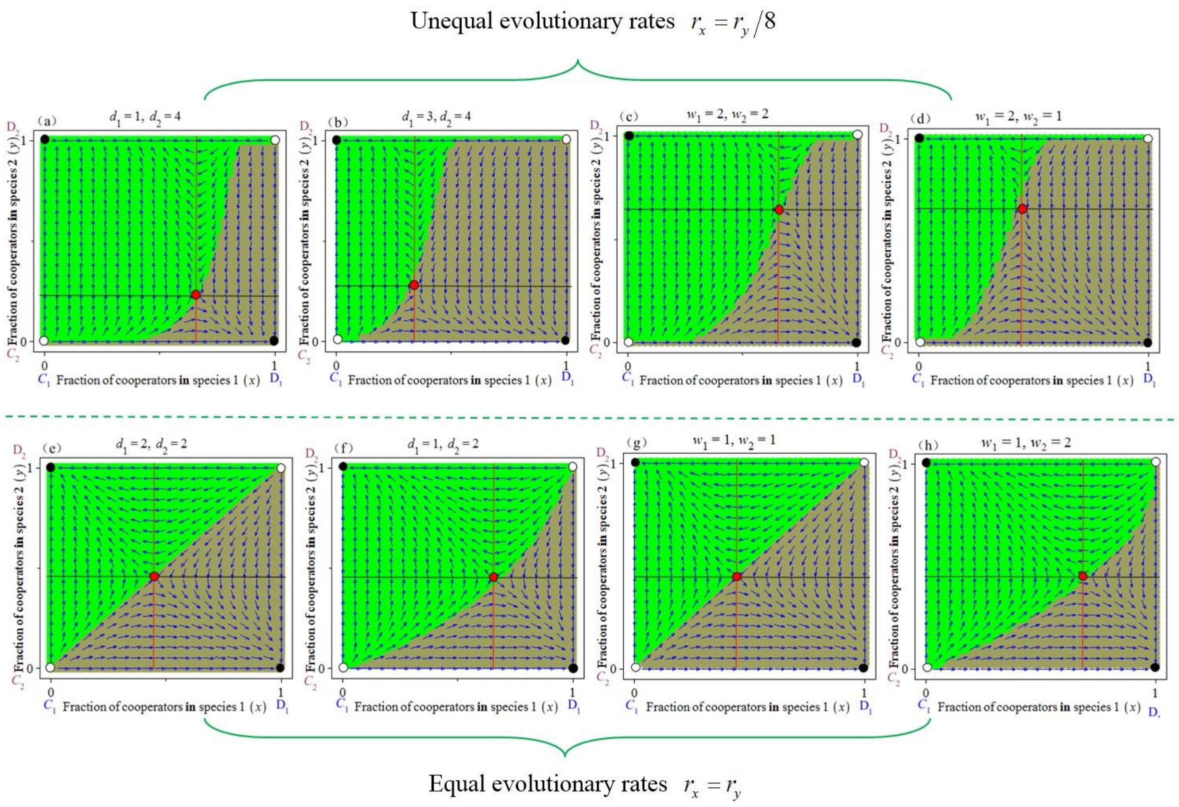

Unequal evolutionary rates $r_x = r_y/8$
(a) $d_1 = 1$, $d_2 = 4$
(b) $d_1 = 3$, $d_2 = 4$
(c) $w_1 = 2$, $w_2 = 2$
(d) $w_1 = 2$, $w_2 = 1$
(e) $d_1 = 2$, $d_2 = 2$
(f) $d_1 = 1$, $d_2 = 2$
(g) $w_1 = 1$, $w_2 = 1$
(h) $w_1 = 1$, $w_2 = 2$
Fraction of cooperators in species 2 (y)
Fraction of cooperators in species 1 (x)
$D_2$
$C_2$
$C_1$
$D_1$
Equal evolutionary rates $r_x = r_y$
